# The Italian Version of the Inventory of Interpersonal Problems (IIP-32): Psychometric Properties and Factor Structure in Clinical and Non-clinical Groups

**DOI:** 10.3389/fpsyg.2018.00341

**Published:** 2018-03-19

**Authors:** Gianluca Lo Coco, Giuseppe Mannino, Laura Salerno, Veronica Oieni, Carla Di Fratello, Gabriele Profita, Salvatore Gullo

**Affiliations:** ^1^Department of Psychology and Educational Sciences, University of Palermo, Palermo, Italy; ^2^Department of Law, LUMSA University, Palermo, Italy; ^3^Faculty of Psychology, University Niccolò Cusano, Rome, Italy

**Keywords:** Inventory of Interpersonal Problems, cross-cultural validity, exploratory structural equation modeling, eating disorders, obesity, psychometric properties

## Abstract

All versions of the Inventory of Interpersonal Problems (IIP) are broadly used to measure people's interpersonal functioning. The aims of the current study are: (a) to examine the psychometric properties and factor structure of the Italian version of the Inventory of Interpersonal Problems—short version (IIP-32); and (b) to evaluate its associations with core symptoms of different eating disorders. One thousand two hundred and twenty three participants (*n* = 623 non-clinical and *n* = 600 clinical participants with eating disorders and obesity) filled out the Inventory of Interpersonal Problems—short version (IIP-32) along with measures of self-esteem (Rosenberg Self-Esteem Scale, RSES), psychological functioning (Outcome Questionnaire, OQ-45), and eating disorders (Eating Disorder Inventory, EDI-3). The present study examined the eight-factor structure of the IIP-32 with Confirmatory Factor Analysis (CFA) and Exploratory Structural Equation Modeling (ESEM). ESEM was also used to test the measurement invariance of the IIP-32 across clinical and non-clinical groups. It was found that CFA had unsatisfactory model fit, whereas the corresponding ESEM solution provided a better fit to the observed data. However, six target factor loadings tend to be modest, and ten items showed cross-loadings higher than 0.30. The configural and metric invariance as well as the scalar and partial strict invariance of the IIP-32 were supported across clinical and non-clinical groups. The internal consistency of the IIP-32 was acceptable and the construct validity was confirmed by significant correlations between IIP-32, RSES, and OQ-45. Furthermore, overall interpersonal difficulties were consistently associated with core eating disorder symptoms, whereas interpersonal styles that reflect the inability to form close relationships, social awkwardness, the inability to be assertive, and a tendency to self-sacrificing were positively associated with general psychological maladjustment. Although further validation of the Italian version of the IIP-32 is needed to support these findings, the results on its cross-cultural validity are promising.

## Introduction

Given the growing recognition of the importance of targeting interpersonal difficulties as outcome variables in psychotherapy, the need for measures of interpersonal problems is warranted. Dysfunctional interpersonal styles are an important dysfunctional area of many psychiatric disorders (Petty et al., 2004; Hilsenroth et al., 2007; Arcelus et al., 2012), and a standardized inventory of typical interpersonal problems can help clients and therapists to determine which problems have been discussed and to specify what has been achieved through treatment.

**Figure 1:**
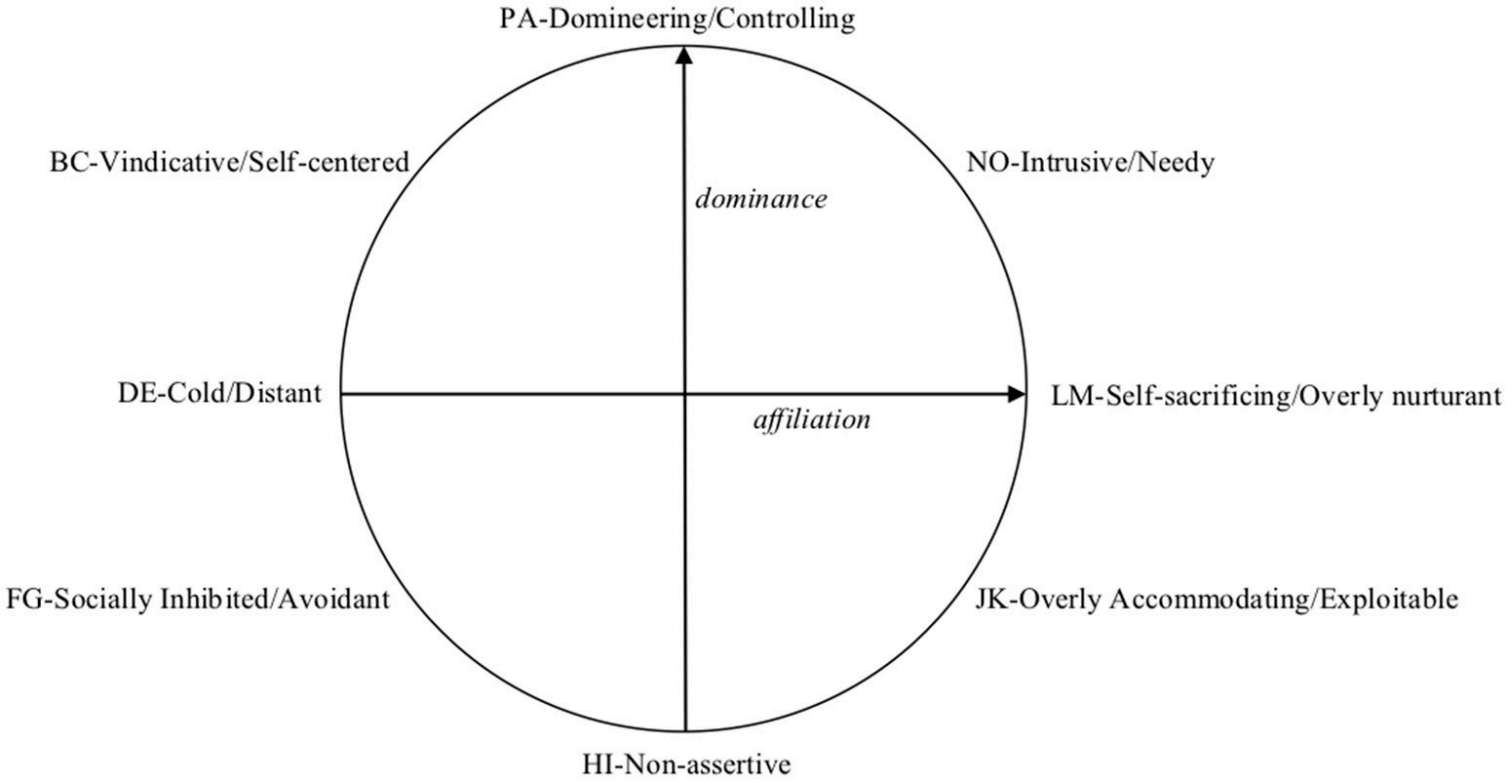
The interpersonal problems circumplex.

The Inventory of Interpersonal Problems (IIP; Horowitz et al., 1988, 2000) is one of the most widely used measures to assess people's interpersonal functioning. It identifies a person's most salient interpersonal difficulties (Horowitz et al., 1988), which are conceptually organized along the dimensions of dominance and affiliation (see Figure 1). The interpersonal characteristics are divided into eight sub-spaces (octants) which are labeled as Domineering/Controlling, Vindictive/ Self-centered, Cold/ Distant, Socially Inhibited/Avoidant, Non-assertive, Overly Accommodating/Exploitable, Self-sacrificing/Overly nurturant, and Intrusive/Needy (Alden et al., 1990).

Since its development, different versions of the IIP were tested and used in clinical research (see Hughes and Barkham, 2005 for a review). In the IIP manual (Horowitz et al., 2000), two versions of the IIP are reported: the 64-item version (IIP-64), and a shorter 32-item version (IIP-32). Both the IIP-64 and the IIP-32 comprise the eight interpersonal dimensions and are used to measure individual's interpersonal distress. The primary spur for the development of a short version (32-items) was to increase the feasibility of it being used as a screening measure in a clinical setting (i.e., to provide a more rapid assessment).

Several additional versions of the IIP have been developed in the last 25 years. Some of them are circumplex versions of the Inventory of Interpersonal Problems (IIP-C, Alden et al., 1990; IIP-SC, Soldz et al., 1995) which use three circumplex summary scores, whereas other versions were based on the factor-analytic approach, such as the IIP-32 by Barkham et al. (1996), the Inventory of Interpersonal Problems-Personality Disorders (IIP-PD; Pilkonis et al., 1996; Kim et al., 1997), and the Inventory of Interpersonal Problems-48 (IIP-48; Gude et al., 2000).

In the current study, we adopted the shorter 32-item scale (IIP-32) by Horowitz et al. (2000), which was developed with the aim of reducing the burden of time in the assessment phase. It is noteworthy that this version of the IIP-32 (Horowitz et al., 2000) differs from the IIP-SC version (Soldz et al., 1995) since five scales differ by one or two items, whereas only three scales (Socially Inhibited/Avoidant, Non-assertive, and Intrusive/Needy) contain the same items as those in the IIP-SC (Horowitz et al., 2000). Furthermore, the IIP-32 by Horowitz et al. (2000) differs from the IIP-32 by Barkham et al. (1996), with the latter sharing only 15 items of the original IIP-32 version by Horowitz (Hughes and Barkham, 2005).

Despite the plethora of IIP measures developed from the interpersonal model described above, psychometric research on the instrument both in English and non-English-speaking communities has repeatedly demonstrated its validity and reliability. Both Alden et al. (1990) and Horowitz et al. (2000) reported acceptable internal consistency reliability values, ranging from alpha = 0.72 to 0.85 for the IIP-64, and also good test-retest reliability (range from *r* = 0.50 to 0.84) (Horowitz et al., 2000). The validity of the IIP has been supported in examinations of its associations with different forms of mental disorders, such as anxiety (Salzer et al., 2008), depression (Grosse Holtforth et al., 2014), eating disorders (Hartmann et al., 2010), as well as with psychological distress and global functioning (Horowitz et al., 2000; Vittengl et al., 2003; Vanheule et al., 2006; McEvoy et al., 2013), insecure attachment (Haggerty et al., 2009), and low self-esteem (Salerno et al., 2015). However, psychometric research on the IIP-32 is still a matter of debate (Horowitz et al., 2000). Previous research showed that both the internal consistency and the factorial structure of the IIP-32 is less satisfactory than the IIP-64 version (Horowitz et al., 2000; Vanheule et al., 2006; Salazar et al., 2010), and a clear eight-factor solution of the IIP-32 was only partially supported (Vanheule et al., 2006; Salazar et al., 2010; McEvoy et al., 2013). However, none of the previous studies on the factorial solution of the IIP adopted the Exploratory Structural Equation Modeling (ESEM), which is particularly useful when analyzing psychological measures with a lack of pure factorial structure (Asparouhov and Muthén, 2009). The current study aims to provide some evidence of the cross-cultural generalizability of the psychometric properties of the IIP-32 to the Italian population, by testing it with both clinical and non-clinical samples.

Most of the research on the psychometric properties of the IIP was conducted with specific clinical groups, given the importance of an individual's interpersonal distress across a wide range of psychiatric disturbances. For example, there is a growing evidence showing the burden of interpersonal distress in individuals with an eating disorder (Wilfley et al., 2005). Hopwood et al. (2007) found that interpersonal dysfunction and bulimic features are mutually influential. Hartmann et al. (2010) found that patients with anorexia and bulimia nervosa exhibited a generally non-assertive, submissive interpersonal style. Lo Coco et al. (2012) found that overweight patients reported elevated interpersonal distress, but these participants were not homogeneous with regard to interpersonal problems. In recent years, the patient's interpersonal distress is growing in importance as a key variable both in the interpersonal model (Arcelus et al., 2013) and in the cognitive interpersonal model (Treasure and Schmidt, 2013) of eating disorders, and research is still exploring the role of interpersonal problems in the development and maintenance of eating disorders.

However, the empirical literature concerning the interpersonal functioning of patients with eating disorder psychopathology is limited by the amount of studies in this area (Arcelus et al., 2013). Most of these studies used non-clinical populations and did not compare clinical and non-clinical groups in the same study. The current study is the first to apply ESEM approaches to testing measurement invariance of the IIP-32 across clinical and non-clinical groups, by focusing on strong and strict factorial/measurement invariance (Marsh et al., 2009), in order to test mean differences based on a latent construct are not due to differential item functioning.

The aim of this study, therefore, were: first, to examine the cross-cultural generalizability of the psychometric properties of the IIP-32 (Horowitz et al., 2000) with two Italian samples. To address this aim we will assess the eight-factor structure of the Italian version of the IIP-32 with CFA and ESEM. Moreover, the present study aims to test the measurement invariance of IIP-32 across clinical and non-clinical groups. It is expected that the strong and strict factorial/measurement invariance (Marsh et al., 2009) of the IIP-32 will be confirmed.

Secondly, the internal consistency of the IIP-32 and its construct validity will be examined. It is expected that patients with eating disorders and obesity would report significantly higher interpersonal difficulties than non-clinical participants; and that high IIP-32 scores will be associated with high psychological distress and low self-esteem levels, consistently with the previous studies on the validity of the IIP (Horowitz et al., 2000; Vanheule et al., 2006; McEvoy et al., 2013; Salerno et al., 2015) in both the clinical and non-clinical samples.

Finally, the present study aims to evaluate the associations of the IIP-32 with core symptoms of different eating disorders (i.e., Anorexia Nervosa, Bulimia Nervosa, Binge Eating Disorder or Night Eating Disorder, and Unspecified Feeding or Eating Disorder). It was hypothesized that the IIP-32 scores will be related to core ED symptoms in patients with an eating disorder, according to the transdiagnostic model (Fairburn, 1997), which suggested that patients with eating disorders have many features in common, including interpersonal distress.

## Methods

### Participants

Two independent samples completed the Italian version of the IIP-32. The first sample was a non-clinical group of *N* = 623 participants ranging from 23 to 67 years of age (Mean = 30.32; *SD* = 9.01; 68% females). The second sample was a clinical group and comprised *N* = 600 participants with obesity (*n* = 297) and Eating Disorders (DSM-5): anorexia nervosa (*n* = 42), bulimia nervosa (*n* = 63), Binge Eating Disorder or Night Eating Disorders (*n* = 143), and Unspecified Feeding or Eating Disorder (*n* = 55). Their ages ranged from 20 to 58 (Mean = 39.81; *SD* = 11.01; 83% females). A sub-sample of 897 participants (*N* = 601 clinical and *N* = 296 non-clinical participants) also completed measures of self-esteem and psychological functioning and a sub-sample of 182 clinical participants with eating disorders (age: Mean = 36.42; *SD* = 10.31; 90.7% females) completed the Eating Disorders Inventory-third edition (Garner, 2004). The distribution of diagnosis of this ED sub-sample was almost the same as the overall ED sample (12.6% anorexia nervosa; 22.0% bulimia nervosa; 51.7% Binge Eating Disorder or Night Eating Disorders; and 13.7% Unspecified Feeding or Eating Disorder).

### Measures

#### Interpersonal difficulties

The IIP-32 (Horowitz et al., 2000) is a 32-item inventory of distressing interpersonal behaviors the respondent identifies as “hard to do” (i.e., behavioral inhibitions) or “does too much” (i.e., behavioral excesses) on a 0 (not at all) to 4 (extremely) Likert-type scale. It provides an overall score and 8 subscale scores (Figure 1): Domineering/Controlling (PA), i.e., being too controlling or manipulative in interpersonal interactions; Vindictive/ Self-centered (BC), i.e., being frequently egocentric and hostile in dealing with others; Cold/ Distant (DE), i.e., having minimal feelings of affection for, and little connection with, other people; Socially Inhibited/Avoidant (FG), i.e., being socially avoidant and anxious, and having difficulty approaching others; Non-assertive (HI), i.e., having difficulty expressing one's needs to others; Overly Accommodating/Exploitable (JK) i.e., being gullible and easily taken advantage of by people; Self-sacrificing/Overly nurturant (LM), i.e., being excessively selfless, generous, trusting, caring, and permissive in dealing with others; and Intrusive/Needy (NO), i.e., imposing one's needs and having difficulty respecting the personal boundaries of other people.

Preserving the scale structure of the 64-item version, the four items of each scale with the highest item-total correlations constituted the shortened version of that scale (Horowitz et al., 2000).

In this study, the Italian translation of the IIP-32 was carried out by a translation method labeled “parallel” or “committee” translation. An advantage of the procedure employed is that it enables structured comparisons between the results of independent translators and incongruence can be resolved or taken to a third part (Harkness and Schoua-Glusberg, 1998). First, two experienced researchers translated the inventory independently. Secondly, the two independent translations were compared, and differences in the translations were discussed until consensus was reached. Thirdly, the consensus version of the translation was given to two experienced psychotherapists and to a researcher in clinical psychology for feedback, and on the basis of their feedback, the instrument was revised a final time. Finally, this final version of the IIP was administered to 25 native Italian university students (12 from the South and 13 from the North) by an email survey in order to check if they saw major flaws in the questionnaire. No critical feedback or request for making item modifications were highlighted.

#### Self-esteem

The Rosenberg Self-esteem Scale (RSES; Rosenberg, 1979) is a widely used self-report 10-item questionnaire of individual's self-esteem. Responses were made on scales ranging from 1 (strongly disagree) to 4 (strongly agree). A higher score indicates more positive self-esteem. This instrument has shown good internal consistency and validity. In the current study, the Italian version of the RSES was used (Salerno et al., 2017), and its Cronbach's alpha was 0.828 and 0.854 for clinical and non-clinical groups, respectively.

#### Psychological functioning

The Outcome Questionnaire-45 (OQ-45; Lambert et al., 2004) is a 45-item self-report questionnaire developed for the purpose of assessing individual's psychological distress (symptoms of distress, social role functioning, and interpersonal relationships) that are of central interest in mental health. The OQ-45 is scored using a five-point Likert-type scale (from 0 “never” to 4 “almost always”), which yields a possible range of scores from 0 to 180. High scores on the OQ indicate more distress. For the purpose of this study only the global score was used. The Italian version of the instrument showed good psychometric properties (Chiappelli et al., 2008; Lo Coco et al., 2008). In the current study, its Cronbach's alpha was 0.919 and 0.935 for clinical and non-clinical groups, respectively.

#### Eating disorders

The Eating Disorder Inventory–third Edition (EDI-3; Garner, 2004) is a widely used 91-item self-report measure of symptomatology associated with eating disorders. This questionnaire has shown good psychometric properties (Garner, 2004). For each item, participants were required to indicate the frequency of their concern or behavior on a six-point Likert-type:: always (0), usually (0), often (1), sometimes (2), rarely (3), and never (4). Higher scores indicated higher likelihood of eating disorders. In this study, only two second-order composite scales of EDI-3 were used (i.e., Eating Disorder Risk Composite—EDRC and General Psychological Maladjustment Composite—GPMC), since those summarize all other subscales and previous studies showed that composite scores represent theoretically distinct higher order constructs (Cumella, 2006). The EDI-3 Cronbach's alpha coefficients were 0.869 and 0.933 for EDRC and GPMC composite, respectively.

### Procedures

The clinical sample was recruited consecutively from two Italian public centers, specialized in the treatment of Eating Disorders and obesity. Patients completed the questionnaires for the study before the beginning of the treatment, as part of their intake assessment, with a paper-and-pencil administration. All participants in the clinical group were assessed by well-experienced clinicians from the two clinics with a structured interview based on the DSM-V criteria for eating disorders (American Psychiatric Association, 2013). Two hundred and ninety-seven patients only reported an obesity condition (with a BMI>30) without an additional diagnosis of bulimia or binge eating. Given the purposes of the current study, they were included in the clinical sample given that they all sought psychosocial treatment for the stress due to their overweight condition.

The non-clinical sample was recruited among university students, friends/relatives of university students and via internet announcements. Ninety-nine non-clinical participants (15.9% of the non-clinical group) filled in an online version of the Inventory of Interpersonal Problems. Five hundred twenty-four (84.1%) non-clinical participants filled out a paper-and-pencil version of the IIP-32. No differences were found between non-clinical participants who compiled the paper-an-pencil version of the questionnaires and those who compiled the online version of them on IIP-32 total score as well as on the majority of IIP-32 subscales (except for FG, LM, and NO subscales) (data not shown). Consequently, both versions of the questionnaires were used in the present study.

The Ethics Committee of University of Palermo (Palermo) approved the study. Both for clinical and non-clinical groups participation in the study was voluntary and participants received no compensation. All participants gave written informed consent.

### Data analysis

Descriptive statistics (mean and standard deviations) were examined. Convergent validity was established by using Spearman's correlation coefficient between all eight subscales and the total score of the IIP-32 scale and standardized measures of self-esteem (RSES) and general psychological functioning (OQ-45). Independent sample *t*-tests were conducted on IIP-32 subscales and the total score to examine differences between clinical and non-clinical participants. Analyses were conducted using PASW (version 17.0).

The eight-factor model of the IIP-32 was examined through the comparison of CFA and ESEM model, as recommended by Marsh et al. (2009). Both in clinical and non-clinical groups, skewness and kurtosis values were analyzed for each variable in order to investigate univariate normality. Multivariate normality was determined through examination of Mardia's test of fit. Regarding univariate normality, some variables exhibited non-normality (skewness ranging from −0.628 to 2.082 and from −0.204 to 2.172, for clinical and non-clinical groups, respectively; kurtosis ranging from −1.175 to 3.994 and from −0.943 to 4.511, for clinical and non-clinical groups, respectively). Also the assumption of multivariate normality was violated (Mardia's coefficient: 73.17 and 45.19 for clinical and non-clinical groups, respectively). Robust maximum likelihood estimator (MLR), which provides tests of model fit and standard errors that are robust to the non-normality of the data and the Likert-type nature of the items, was used. The items were treated as continuous variables. Moreover, the measurement invariance across clinical and non-clinical samples was evaluated. Firstly, the model fit for each group was separately assessed (Sass, 2011) and, secondly, several levels of group invariance (Cheung and Rensvold, 2002) were tested: unconstrained based model (M0—configural invariance), factor loadings (M1—metric invariance), item intercepts (M2—scalar invariance), residuals variances (M3—strict variance), factor variances and covariances (M4), and factor means (M5). CFI decreases ≥ 0.010 are considered as indicators of meaningful differences across groups (Cheung and Rensvold, 2002; Chen, 2007). All analyses were performed by Mplus version 6.12 (Muthén and Muthén, 1998-2012).

Conventional fit indices were used to evaluate the overall model goodness fit: the χ^2^ test statistics (χ^2^/df ratios < 3 indicate reasonable fitting models), the comparative fit index (CFI) and Tucker-Lewis Index (TLI) (with values close to 0.95 indicating better fitting models), the root-mean-square error of approximation (RMSEA) and the Standardized Root Mean Squared Residuals (SRMR) (with values of 0.05 or less indicating close fit) (Knight et al., 1994; Hoyle and Panter, 1995; Hu and Bentler, 1999; Marsh et al., 2004; Byrne, 2008).

Reliability was estimated using the following indices of the latent factors: the omega/omega subscale coefficient (ω)/(ωS), which estimates the proportion of variance in the observed total score/subscale score attributable to all modeled sources of common variance; the omega hierarchical coefficient (ωH), which estimates the proportion of variance in total scores that can be attributed to a single general factor after accounting for the specific factors; the omega hierarchical subscale coefficient (ωHS), which estimates the proportion of reliable systematic variance associated with each specific factor, after partialling out variability accounted for the general factor (Reise et al., 2013). When ωH is above 0.80 total score should be considered essentially unidimensional, an ωHS > 0.75 indicates that the subscale score in question is an appropriate measure of its corresponding specific factor. Moreover, reliability both for CFA and ESEM eight factors was estimated by using factor score determinacy coefficients, which represent an estimate of the internal consistency of the factor solution (Tabachnick and Fidell, 2013). The larger the coefficient (≥0.70), the more stable the factors (Tabachnick and Fidell, 2013).

Finally, a series of hierarchical regression analyses were conducted where EDI-3 composite scores were regressed on the IIP-32 subscales and total score.

## Results

### Item analysis

Item correlations, means, standard deviations skewness and kurtosis for the IIP-32 items (both for clinical and non-clinical groups) are displayed in Table 1.

**Table 1:** Correlations and descriptives for the IIP-32 items.

	**IIP1**	**IIP2**	**IIP3**	**IIP4**	**IIP5**	**IIP6**	**IIP7**	**IIP8**	**IIP9**	**IIP10**	**IIP11**	**IIP12**	**IIP13**	**IIP14**	**IIP15**	**IIP16**	**IIP17**	**IIP18**	**IIP19**	**IIP20**	**IIP21**	**IIP22**	**IIP23**	**IIP24**	**IIP25**	**IIP26**	**IIP27**	**IIP28**	**IIP29**	**IIP30**	**IIP31**	**IIP32**
IIP1	–	0.233^**^	0.247^**^	0.443^**^	0.212^**^	0.219^**^	0.209^**^	0.219^**^	0.150^**^	0.084^*^	0.004	0.346^**^	0.070	0.028	0.029	−0.049	−0.089^*^	−0.018	0.094^*^	0.219^**^	0.191^**^	−0.011	0.150^**^	0.007	0.075	0.340^**^	0.261^**^	0.007	0.175^**^	0.015	0.375^**^	0.160^**^
IIP2	0.176^**^	–	0.197^**^	0.299^**^	0.573^**^	0.412^**^	0.319^**^	0.248^**^	0.624^**^	0.270^**^	0.338^**^	0.308^**^	0.226^**^	0.203^**^	0.212^**^	0.152^**^	0.125^**^	0.194^**^	0.455^**^	0.255^**^	−0.057	0.044	0.115^**^	0.043	0.096^*^	0.136^**^	0.056	0.077	0.088^*^	0.024	0.176^**^	0.083^*^
IIP3	0.202^**^	0.135^**^	–	0.295^**^	0.156^**^	0.193^**^	0.245^**^	0.113^**^	0.130^**^	0.037	0.082^*^	0.239^**^	0.045	0.132^**^	0.021	0.044	0.017	0.141^**^	0.089^*^	0.156^**^	0.377^**^	0.120^**^	0.252^**^	0.135^**^	0.168^**^	0.213^**^	0.176^**^	0.151^**^	0.547^**^	0.211^**^	0.309^**^	0.116^**^
IIP4	0.469^**^	0.259^**^	0.300^**^	–	0.295^**^	0.400^**^	0.318^**^	0.277^**^	0.231^**^	0.169^**^	0.039	0.410^**^	0.072	0.109^**^	0.067	0.054	0.009	0.071	0.197^**^	0.281^**^	0.194^**^	−0.056	0.254^**^	0.071	0.095^*^	0.311^**^	0.193^**^	0.076	0.250^**^	0.020	0.341^**^	0.212^**^
IIP5	0.188^**^	0.550^**^	0.106^**^	0.263^**^	–	0.460^**^	0.379^**^	0.154^**^	0.646^**^	0.308^**^	0.256^**^	0.294^**^	0.189^**^	0.164^**^	0.184^**^	0.070	0.090^*^	0.168^**^	0.443^**^	0.258^**^	0.019	0.080^*^	0.112^**^	0.007	0.116^**^	0.168^**^	0.112^**^	0.048	0.145^**^	0.093^*^	0.219^**^	0.114^**^
IIP6	0.199^**^	0.451^**^	0.134^**^	0.295^**^	0.583^**^	–	0.410^**^	0.295^**^	0.412^**^	0.255^**^	0.255^**^	0.424^**^	0.210^**^	0.278^**^	0.176^**^	0.202^**^	0.057	0.181^**^	0.360^**^	0.313^**^	0.090^*^	0.133^**^	0.253^**^	0.165^**^	0.118^**^	0.213^**^	0.090^*^	0.184^**^	0.207^**^	0.089^*^	0.323^**^	0.238^**^
IIP7	0.241^**^	0.300^**^	0.107^**^	0.231^**^	0.283^**^	0.391^**^	–	0.226^**^	0.309^**^	0.285^**^	0.217^**^	0.325^**^	0.208^**^	0.226^**^	0.203^**^	0.152^**^	0.121^**^	0.203^**^	0.271^**^	0.383^**^	0.063	0.118^**^	0.153^**^	0.036	0.128^**^	0.197^**^	0.125^**^	0.124^**^	0.185^**^	0.140^**^	0.263^**^	0.154^**^
IIP8	0.188^**^	0.204^**^	0.115^**^	0.285^**^	0.190^**^	0.204^**^	0.223^**^	–	0.187^**^	0.211^**^	0.195^**^	0.232^**^	0.138^**^	0.187^**^	0.243^**^	0.203^**^	0.092^*^	0.173^**^	0.157^**^	0.107^**^	0.010	−0.016	0.132^**^	0.082^*^	0.176^**^	0.134^**^	0.085^*^	0.155^**^	0.054	0.044	0.168^**^	0.097^*^
IIP9	0.104^*^	0.638^**^	0.060	0.176^**^	0.650^**^	0.485^**^	0.285^**^	0.148^**^	–	0.377^**^	0.386^**^	0.238^**^	0.226^**^	0.240^**^	0.227^**^	0.216^**^	0.182^**^	0.269^**^	0.559^**^	0.220^**^	−0.084^*^	0.134^**^	0.121^**^	0.056	0.094^*^	0.069	0.066	0.129^**^	0.081^*^	0.085^*^	0.167^**^	0.090^*^
IIP10	0.039	0.301^**^	0.096^*^	0.088^*^	0.321^**^	0.324^**^	0.179^**^	0.092^*^	0.443^**^	–	0.395^**^	0.166^**^	0.408^**^	0.252^**^	0.408^**^	0.204^**^	0.116^**^	0.303^**^	0.333^**^	0.214^**^	−0.181^**^	0.170^**^	0.066	0.067	0.180^**^	0.043	−0.058	0.199^**^	−0.011	0.132^**^	0.033	0.053
IIP11	−0.043	0.437^**^	0.108^**^	0.025	0.377^**^	0.290^**^	0.231^**^	0.074	0.482^**^	0.462^**^	–	0.237^**^	0.332^**^	0.430^**^	0.433^**^	0.378^**^	0.220^**^	0.232^**^	0.333^**^	0.154^**^	−0.081^*^	0.403^**^	0.136^**^	0.187^**^	0.284^**^	0.008	−0.064	0.235^**^	0.030	0.254^**^	0.145^**^	0.096^*^
IIP12	0.318^**^	0.244^**^	0.196^**^	0.407^**^	0.289^**^	0.310^**^	0.294^**^	0.216^**^	0.232^**^	0.217^**^	0.180^**^	–	0.167^**^	0.250^**^	0.168^**^	0.104^**^	0.036	0.051	0.221^**^	0.277^**^	0.153^**^	0.090^*^	0.187^**^	0.098^*^	0.106^**^	0.258^**^	0.155^**^	0.108^**^	0.235^**^	0.121^**^	0.358^**^	0.220^**^
IIP13	0.074	0.250^**^	0.058	0.088^*^	0.202^**^	0.205^**^	0.153^**^	0.057	0.273^**^	0.331^**^	0.328^**^	0.210^**^	–	0.369^**^	0.423^**^	0.272^**^	0.156^**^	0.269^**^	0.196^**^	0.019	−0.016	0.238^**^	0.081^*^	0.104^**^	0.186^**^	0.047	−0.020	0.148^**^	0.058	0.176^**^	0.077	0.057
IIP14	0.001	0.214^**^	0.112^**^	0.089^*^	0.206^**^	0.266^**^	0.143^**^	0.062	0.250^**^	0.291^**^	0.432^**^	0.248^**^	0.301^**^	–	0.479^**^	0.455^**^	0.293^**^	0.266^**^	0.184^**^	0.129^**^	0.040	0.239^**^	0.131^**^	0.124^**^	0.213^**^	0.057	0.017	0.226^**^	0.120^**^	0.223^**^	0.136^**^	0.119^**^
IIP15	0.031	0.322^**^	0.032	0.044	0.299^**^	0.312^**^	0.155^**^	0.102^*^	0.419^**^	0.482^**^	0.488^**^	0.202^**^	0.454^**^	0.576^**^	–	0.512^**^	0.267^**^	0.250^**^	0.205^**^	0.111^**^	−0.113^**^	0.238^**^	0.144^**^	0.193^**^	0.228^**^	−0.008	−0.041	0.206^**^	−0.050	0.153^**^	0.047	0.057
IIP16	−0.059	0.179^**^	0.036	−0.003	0.134^**^	0.194^**^	0.110^**^	0.075	0.241^**^	0.300^**^	0.330^**^	0.131^**^	0.242^**^	0.510^**^	0.557^**^	–	0.489^**^	0.320^**^	0.186^**^	0.052	−0.038	0.260^**^	0.173^**^	0.167^**^	0.233^**^	−0.074	−0.043	0.288^**^	0.029	0.182^**^	0.115^**^	0.048
IIP17	−0.122^**^	0.139^**^	0.055	−0.009	0.116^**^	0.141^**^	0.086^*^	0.020	0.185^**^	0.273^**^	0.271^**^	0.105^*^	0.120^**^	0.434^**^	0.371^**^	0.520^**^	–	0.343^**^	0.186^**^	0.049	0.041	0.190^**^	0.123^**^	0.111^**^	0.165^**^	−0.217^**^	−0.103^*^	0.194^**^	0.080^*^	0.174^**^	0.019	−0.093^*^
IIP18	−0.026	0.275^**^	0.087^*^	0.083^*^	0.166^**^	0.252^**^	0.139^**^	0.066	0.242^**^	0.314^**^	0.363^**^	0.192^**^	0.268^**^	0.383^**^	0.407^**^	0.424^**^	0.433^**^	–	0.330^**^	0.113^**^	0.014	0.102^*^	0.108^**^	0.097^*^	0.114^**^	−0.020	0.033	0.191^**^	0.121^**^	0.144^**^	0.095^*^	−0.020
IIP19	0.166^**^	0.480^**^	0.011	0.193^**^	0.558^**^	0.420^**^	0.240^**^	0.191^**^	0.583^**^	0.364^**^	0.386^**^	0.208^**^	0.260^**^	0.222^**^	0.356^**^	0.200^**^	0.163^**^	0.239^**^	–	0.281^**^	−0.109^**^	0.087^*^	0.117^**^	0.058	0.101^*^	0.076	0.028	0.121^**^	0.035	0.136^**^	0.159^**^	0.074
IIP20	0.316^**^	0.132^**^	0.053	0.314^**^	0.192^**^	0.270^**^	0.326^**^	0.238^**^	0.127^**^	0.127^**^	0.038	0.278^**^	0.110^**^	0.084^*^	0.039	0.014	−0.009	0.051	0.249^**^	–	0.094^*^	0.051	0.115^**^	0.107^**^	0.090^*^	0.188^**^	0.091^*^	0.079^*^	0.113^**^	0.061	0.202^**^	0.153^**^
IIP21	0.088^*^	−0.132^**^	0.361^**^	0.149^**^	−0.207^**^	−0.118^**^	0.011	−0.033	−0.288^**^	−0.239^**^	−0.147^**^	0.073	−0.112^**^	−0.083^*^	−0.220^**^	−0.158^**^	−0.126^**^	−0.061	−0.259^**^	0.039	–	0.131^**^	0.171^**^	0.097^*^	0.146^**^	0.238^**^	0.308^**^	0.080^*^	0.612^**^	0.270^**^	0.352^**^	0.145^**^
IIP22	0.003	0.108^**^	0.094^*^	−0.006	0.089^*^	0.098^*^	0.148^**^	−0.124^**^	0.068	0.125^**^	0.303^**^	0.086^*^	0.125^**^	0.142^**^	0.146^**^	0.074	0.150^**^	0.252^**^	0.066	0.042	0.133^**^	–	0.172^**^	0.234^**^	0.316^**^	0.028	−0.003	0.330^**^	0.149^**^	0.453^**^	0.108^**^	0.080^*^
IIP23	0.128^**^	0.119^**^	0.164^**^	.235^**^	0.040	0.129^**^	0.106^**^	0.130^**^	0.005	0.042	0.128^**^	0.209^**^	0.110^**^	0.120^**^	0.097^*^	0.030	0.070	0.150^**^	0.089^*^	0.111^**^	0.238^**^	0.213^**^	–	0.649^**^	0.358^**^	0.192^**^	0.109^**^	0.330^**^	0.271^**^	0.187^**^	0.243^**^	0.297^**^
IIP24	0.058	0.062	0.189^**^	0.124^**^	0.054	0.106^**^	0.111^**^	0.019	0.012	0.064	0.107^**^	0.102^*^	0.037	0.054	0.031	0.018	0.103^*^	0.141^**^	0.041	0.093^*^	0.217^**^	0.325^**^	0.588^**^	–	0.401^**^	0.091^*^	−0.026	0.346^**^	0.193^**^	0.157^**^	0.162^**^	0.263^**^
IIP25	0.024	0.040	0.152^**^	0.145^**^	0.007	0.076	0.020	0.011	0.008	0.127^**^	0.188^**^	0.085^*^	0.156^**^	0.125^**^	0.110^**^	0.076	0.141^**^	0.164^**^	0.073	0.101^*^	0.095^*^	0.369^**^	0.362^**^	0.444^**^	–	0.196^**^	0.068	0.550^**^	0.230^**^	0.368^**^	0.170^**^	0.158^**^
IIP26	0.338^**^	0.106^**^	0.146^**^	0.319^**^	0.163^**^	0.132^**^	0.178^**^	0.163^**^	0.068	−0.066	0.052	0.219^**^	0.111^**^	−0.082^*^	−0.023	−0.202^**^	−0.280^**^	−0.068	0.128^**^	0.219^**^	0.126^**^	0.068	0.223^**^	0.057	0.106^**^	–	0.549^**^	0.073	0.271^**^	0.141^**^	0.414^**^	0.368^**^
IIP27	0.298^**^	0.029	0.166^**^	0.157^**^	0.042	0.024	0.114^**^	0.138^**^	−0.022	−0.123^**^	0.014	0.085^*^	0.032	−0.069	−0.083^*^	−0.165^**^	−0.172^**^	−0.019	0.013	0.128^**^	0.289^**^	0.074	0.183^**^	0.096^*^	0.058	0.471^**^	–	0.024	0.263^**^	0.201^**^	0.362^**^	0.233^**^
IIP28	−0.015	0.006	0.130^**^	0.045	−0.003	0.055	0.025	−0.004	−0.025	0.130^**^	0.108^**^	0.038	0.143^**^	0.176^**^	0.141^**^	0.153^**^	0.193^**^	0.217^**^	−0.020	0.062	0.070	0.290^**^	0.268^**^	0.399^**^	0.492^**^	0.016	−0.026	–	0.191^**^	0.330^**^	0.124^**^	0.077
IIP29	0.110^**^	0.035	0.463^**^	0.212^**^	−0.025	0.062	0.107^**^	−0.022	−0.106^**^	−0.039	−0.007	0.136^**^	−0.001	0.043	−0.043	−0.067	0.017	0.106^**^	−0.039	0.093^*^	0.612^**^	0.271^**^	0.276^**^	0.300^**^	0.183^**^	0.166^**^	0.202^**^	0.193^**^	–	0.331^**^	0.398^**^	0.199^**^
IIP30	0.068	0.013	0.177^**^	0.062	−0.039	−0.059	0.039	−0.107^**^	−0.074	0.021	0.162^**^	0.018	0.093^*^	0.089^*^	0.023	−0.008	0.123^**^	0.115^**^	−0.017	0.029	0.310^**^	0.456^**^	0.200^**^	0.278^**^	0.332^**^	0.072	0.178^**^	0.272^**^	0.406^**^	–	0.242^**^	0.133^**^
IIP31	0.332^**^	0.113^**^	0.207^**^	0.370^**^	0.172^**^	0.193^**^	0.173^**^	0.148^**^	0.088^*^	−0.024	0.045	0.195^**^	0.065	0.030	0.043	−0.073	−0.114^**^	0.028	0.135^**^	0.159^**^	0.218^**^	0.080^*^	0.230^**^	0.135^**^	0.057	0.399^**^	0.452^**^	0.013	0.355^**^	0.203^**^	–	0.384^**^
IIP32	0.277^**^	0.111^**^	0.169^**^	0.289^**^	0.148^**^	0.139^**^	0.173^**^	0.149^**^	0.089^*^	−0.025	0.039	0.153^**^	0.079	0.008	−0.043	−0.140^**^	−0.125^**^	−0.060	0.120^**^	0.240^**^	0.182^**^	0.118^**^	0.218^**^	0.128^**^	0.166^**^	0.450^**^	0.361^**^	0.052	0.229^**^	0.193^**^	0.389^**^	–
M	2.48	1.15	1.37	1.71	1.23	1.43	1.47	1.30	0.85	0.89	0.65	1.48	0.76	0.65	0.51	0.68	0.86	0.58	1.29	2.01	1.87	1.01	1.41	0.78	0.94	1.73	2.29	0.47	1.39	1.27	1.70	1.85
SD	1.201	1.278	1.240	1.412	1.311	1.276	1.197	1.296	1.175	1.244	0.940	1.240	1.170	0.931	0.903	0.987	1.108	1.006	1.340	1.307	1.323	1.148	1.372	1.161	1.169	1.305	1.193	0.873	1.298	1.191	1.302	1.301
Skew	−0.628	0.747	0.511	0.201	0.749	0.473	0.360	0.589	1.225	1.184	1.447	0.442	1.386	1.345	1.799	1.375	1.218	1.694	0.683	0.002	0.089	0.936	0.452	1.257	1.042	0.199	−0.236	2.082	0.554	0.576	0.328	0.135
Kurt	−0.543	−0.733	−0.850	−1.330	−0.686	−0.928	−0.849	−0.896	0.370	0.137	1.446	−0.879	0.708	0.926	2.475	1.058	0.582	1.835	−0.778	−1.175	−1.130	−0.141	−1.162	0.325	−0.023	−1.164	−0.870	3.994	−0.875	−0.769	−1.082	−1.149
M	2.00	0.79	0.86	1.25	0.82	0.92	1.11	1.14	0.57	0.74	0.48	1.05	0.49	0.51	0.36	0.50	0.90	0.41	0.85	1.58	1.44	0.73	1.12	0.72	0.76	1.34	1.89	0.48	0.96	1.05	1.13	1.59
SD	1.138	1.030	1.022	1.159	1.044	0.981	0.972	1.083	0.870	1.012	0.750	0.994	0.890	0.796	0.690	0.746	0.922	0.803	1.046	1.136	1.164	0.910	1.078	1.013	0.966	1.094	1.035	0.824	1.089	1.006	1.091	1.134
Skew	−0.204	1.121	1.011	0.553	1.128	0.901	0.650	0.538	1.566	1.290	1.577	0.700	1.888	1.644	2.034	1.501	0.948	2.172	1.158	0.295	0.298	1.176	0.625	1.307	1.089	0.468	0.065	1.707	0.881	0.722	0.825	0.269
Kurt	−0.756	0.309	0.134	−0.691	0.374	0.154	−0.017	−0.832	1.855	0.867	2.072	−0.204	2.901	2.322	3.956	2.067	0.591	4.511	0.607	−0.706	−0.943	0.823	−0.470	0.876	0.257	−0.568	−0.550	2.103	−0.222	−0.107	0.191	−0.743

*For correlations, the lower half-matrix shows data for the clinical group; the upper half-matrix shows data for the non-clinical group. For descriptives, the upper data refer to the clinical group; the lower data refer to the non-clinical group M, mean; SD, Standard Deviation; IIP, Inventory of Interpersonal Problems; Skew, Skewness; Kurt, Kurtosis*.

* p < 0.05;

** *p < 0.01*.

### Inventory of interpersonal problems factor structure in the clinical sample: CFA vs. ESEM

CFA had unsatisfactory model fit (χ^2^ = 1586.345; df = 436; CFI = 0.798; TLI = 0.770; RMSEA = 0.066; SRMR = 0.078; RMSEA 90% CI = 0.063–0.070), whereas the corresponding ESEM solution provided a better fit to the observed data (χ^2^ = 512.871; df = 268; CFI = 0.957; TLI = 0.920; RMSEA = 0.039; SRMR = 0.021; RMSEA 90% CI = 0.034–0.044), as indicated by higher CFI and lower RMSEA values. Factor correlations between the eight factors were lower in the ESEM model (range:−0.136 to −0.553) than in the CFA model (range: −0.236 to −0.935) (see Table 2).

**Table 2:** Latent factor correlations in clinical group (*n* = 600).

	**PA**	**BC**	**DE**	**FG**	**HI**	**JK**	**LM**	**NO**
PA	–	0.303^***^	0.313^***^	0.031	0.156^*^	0.132	0.289^***^	0.519^***^
BC	0.229^***^	–	0.815^***^	0.380^***^	0.355^***^	−0.030	−0.236^***^	−0.013
DE	0.121	0.553^***^	–	0.687^***^	0.476^***^	0.126^*^	0.000	−0.100
FG	0.077	0.284^***^	0.442^***^	–	0.720^***^	0.376^***^	0.161^**^	−0.128^*^
HI	0.094	0.117	0.144^*^	0.262^**^	–	0.935^***^	0.473^***^	0.254^***^
JK	−0.236^**^	−0.136	0.080	0.163	0.025	–	0.890^***^	0.386^***^
LM	0.240^*^	−0.109	0.037	0.141^**^	0.329^**^	0.180	–	0.421^***^
NO	0.335^***^	0.037	−0.097	−0.035	0.087	0.126	0.400^***^	–

*CFA correlations are displayed above the diagonal and ESEM correlations are displayed below the diagonal. PA, IIP-32 Domineering/Controlling subscale; BC, IIP-32 Vindictive/ Self-centered subscale; DE, IIP-32 Cold/ Distant subscale; FG, Socially Inhibited/Avoidant subscale; HI, IIP-32 Non-assertive subscale; JK, IIP-32 Overly Accommodating/Exploitable subscale; LM, IIP-32 Self-sacrificing/Overly nurturant subscale; NO, IIP-32 Intrusive/Needy subscale*.

* p < 0.05;

** p < 0.01;

*** *p < 0.001*.

Standardized parameter estimates are shown in Table 3. The overall size of the factor loadings of the items on their target factors is higher in the CFA model (λ = 0.347 to 0.847; M = 0.615) than in ESEM (λ = 0.069 to 0.846; M = 0.512). More specifically, in ESEM model target factor loadings of Item 6, Item 7, Item 8, Item 20, Item 24, and Item 31 were lower than 0.30. Looking at the cross-loadings, ten of them were higher than 0.30: Item 24 of Intrusive/Needy factor (“*I want to be noticed too much*”) cross-loaded on the Domineering/Controlling factor at 0.38; Item 14 of Vindictive/Self-centered factor (“*be supportive of another person's goals in life*”) cross-loaded on the Cold/distant factor at 0.337; Item 6 of Non-assertive factor (“*confront people with problems that come up*”) cross loaded on the Socially Inhibited factor at 0.478; Item 11 of Cold/distant factor (“*get along with people*”) cross-loaded on the Socially Inhibited factor at 0.351; Item 1 (“*say no to other people*”), Item 8 (“*let other people know when I am angry*”), and Item 20 (“*be assertive without worrying about hurting the other person's feelings*”) of the Overly-Accommodating factor cross-loaded on the Non-assertive factor at 0.381, 0.307, and 0.400, respectively; Item 23 of the Self-sacrificing factor (“*I try to please other people to much*”) cross-loaded on the Overly-Accommodating factor at −0.440; Item 24 of the Intrusive/needy factor (“*I want to be noticed too much*”) cross-loaded on the Overly-Accommodating factor at −0.442 and Item 31 of the Overly-Accommodating factor (“*I let other people take advantage of me too much*”) cross-loaded on the Self-sacrificing factor at 0.468. Both in CFA and ESEM, all factor scores had high determinacy (Table 3).

**Table 3:** Standardized parameter estimate for the CFA and ESEM solutions of the Inventory of Interpersonal Problems-32 in clinical group (*n* = 600).

**Items**	**CFA**								**ESEM**							
	**PA**	**BC**	**DE**	**FG**	**HI**	**JK**	**LM**	**NO**	**PA**	**BC**	**DE**	**FG**	**HI**	**JK**	**LM**	**NO**
IIP22	**0.627**								**0.610**	0.043	0.051	0.104	0.458	0.104	0.014	0.088
IIP25	**0.619**								**0.656**	−0.020	0.096	−0.110	0.174	−0.054	0.094	−0.087
IIP28	**0.550**								**0.538**	0.080	0.092	−0.161	0.130	−0.059	−0.027	−0.004
IIP30	**0.629**								**0.550**	0.070	−0.027	−0.031	−0.258	0.240	0.057	0.275
IIP14		**0.704**							−0.020	**0.503**	0.337	−0.088	0.040	0.039	0.020	0.045
IIP16		**0.731**							−0.087	**0.678**	0.178	−0.099	0.029	0.000	−0.035	−0.052
IIP17		**0.647**							0.112	**0.803**	−0.196	0.024	0.003	0.052	−0.129	−0.064
IIP18		**0.605**							0.117	**0.488**	0.098	0.071	−0.001	0.015	0.008	0.039
IIP10			**0.617**						0.126	0.040	**0.421**	0.221	0.144	−0.017	−0.220	−0.027
IIP11			**0.696**						0.173	0.142	**0.371**	0.351	−0.181	−0.034	0.072	−0.025
IIP13			**0.502**						0.104	−0.057	**0.593**	−0.032	0.043	0.036	0.074	−0.026
IIP15			**0.774**						−0.073	0.292	**0.667**	−0.010	−0.015	−0.059	0.055	−0.011
IIP2				**0.727**					−0.053	0.022	0.105	**0.659**	0.092	−0.047	0.013	0.103
IIP5				**0.773**					−0.024	−0.014	0.033	**0.729**	0.172	0.069	0.008	–.034
IIP9				**0.847**					−0.066	0.005	0.176	**0.760**	0.010	−0.048	−0.023	−0.070
IIP19				**0.697**					0.016	0.046	0.128	**0.550**	0.122	0.050	0.089	−0.156
IIP4					**0.584**				−0.009	0.017	−0.037	0.056	**0.565**	0.360	0.129	0.225
IIP6					**0.632**				−0.026	0.091	0.057	0.478	**0.291**	0.069	−0.018	0.063
IIP7					**0.503**				0.045	0.112	−0.027	0.252	**0.216**	0.206	0.085	0.043
IIP12					**0.534**				−0.026	0.098	0.135	0.050	**0.423**	0.198	0.055	0.153
IIP1						**0.555**			−0.007	−0.045	−0.001	−0.010	0.458	**0.336**	0.271	0.009
IIP8						**0.346**			−0.167	0.126	−0.037	0.064	0.397	**0.069**	0.257	−0.083
IIP20						**0.442**			0.128	0.017	0.041	0.038	0.523	**0.279**	0.080	−0.073
IIP31						**0.569**			−0.068	0.025	0.000	0.072	0.017	**0.200**	0.609	0.278
IIP23							**0.357**		0.128	0.085	0.014	−0.029	0.285	−0.604	**0.483**	0.183
IIP26							**0.704**		0.033	−0.274	0.170	0.007	0.062	0.288	**0.562**	−0.049
IIP27							**0.619**		−0.052	0.044	−0.088	−0.001	−0.273	0.244	**0.706**	0.031
IIP32							**0.627**		0.138	−0.104	−0.009	0.067	0.063	0.310	**0.412**	0.042
IIP3								**0.526**	−0.018	0.004	0.074	0.055	0.217	0.057	−0.094	**0.556**
IIP21								**0.713**	−0.051	−0.037	−0.090	−0.161	−0.036	−0.036	0.078	**0.724**
IIP24								**0.393**	0.380	0.049	−0.126	0.082	0.203	−0.512	0.286	**0.135**
IIP29								**0.839**	0.087	−0.027	0.018	0.016	0.022	0.021	−0.097	**0.846**
**FS DETERMINACY**																
	0.863	0.911	0.927	0.938	0.923	0.945	0.888	0.905	0.888	0.910	0.898	0.930	0.851	0.834	0.895	0.915

*PA, IIP-32 Domineering/Controlling subscale; BC, IIP-32 Vindictive/ Self-centered subscale; DE, IIP-32 Cold/ Distant subscale; FG, IIP-32 Socially Inhibited/Avoidant subscale; HI, IIP-32 Non-assertive subscale; JK, IIP-32 Overly Accommodating/Exploitable subscale; LM, IIP-32 Self-sacrificing/Overly nurturant subscale; NO, IIP-32 Intrusive/Needy subscale. Bold characters indicate factor loadings of items on their target factors*.

### The measurement invariance across clinical and non-clinical groups

The eight-factor ESEM model had a good fit both in clinical and non-clinical groups (Table 4). The configural model (M0) fit was good. For metric invariance (model M1) the comparison of M1 vs. M0 showed a significant χ^2^ difference test but worsening of CFI did not exceeded the threshold (ΔCFI < 0.010), showing no differences for factor loadings among the two groups. Also scalar invariance (model M2) was satisfactory in terms of model fit and relative change in fit. The comparison of M3 vs. M2 (strict invariance) showed a significant scaled χ^2^ difference test and ΔCFI exceeded the threshold (>0.010), suggesting differential residual variances. The model was therefore modified releasing the equality constraint imposed on the residual variances of Items 3, 5, 6, 7, 9, 10, 12, 13, 16, 22, 23, 25, 26, and 31. The comparison of the modified model M3a with M2 showed a significant χ^2^ difference test, but worsening of CFI did not exceed the threshold, suggesting a partial strict invariance. Finally, the comparisons M4 and M3a and of M5 and M4 were unsatisfactory in terms of relative change in fit, suggesting that latent variances/covariances and latent means invariance were not achieved.

**Table 4:** IIP-32 eight-factor ESEM model invariance across clinical and non-clinical groups.

	**χ^2^**	**df**	**CFI**	**TLI**	**RMSEA**	**SRMR**	**Model comparison**	**Δ*χ*^2^**	**Δdf**	**ΔCFI**
Non-clinical group	553.276	268	0.949	0.905	0.041	0.023	–	–	–	–
Clinical group	512.871	268	0.957	0.920	0.039	0.021	–	–	–	–
M0—configural invariance	1070.258	536	0.953	0.912	0.040	0.022	–	–	–	–
M1—metric invariance	1258.826	728	0.953	0.936	0.035	0.032	M1-M0	188.568^***^	192	0.000
M2—scalar invariance	1325.023	752	0.949	0.933	0.035	0.032	M2-M1	66.197^***^	24	0.004
M3—strict invariance	1858.295	784	0.905	0.879	0.047	0.046	M3-M2	533.272^***^	32	0.044
M3a—partial strict invariance	1449.867	770	0.940	0.922	0.038	0.037	M3a-M2	124.844^***^	18	0.009
M4—factor variances/covariances invariance	1637.092	806	0.926	0.909	0.041	0.059	M4-M3a	187.225^***^	36	0.014
M5—factor means invariance	1765.783	814	0.915	0.897	0.044	0.064	M5-M4	128.691^***^	8	0.011

*df, degree of freedom; CFI, Comparative fit index; TLI, Tucker-Lewis Index; RMSEA, Root-mean-square error of approximation; SRMR, Standardized Root Mean Squared Residuals*.

*** *p < 0.001*.

### Internal consistency

In the non-clinical sample, the omega coefficient (ω) for total score was 0.985; whereas the ωS for the eight scores ranged from 0.776 (Factor 6) to 0.975 (Factor 2). The hierarchical omega coefficient (ωH) for the total score was 0.874, whereas the ωHS for the eight subscales ranged from 0.388 to 0.487. In the clinical sample, the omega coefficient (ω) for total score was 0.983; whereas the ωS for the eight scores ranged from 0.771 (Factor 6) to 0.946 (Factor 8). The hierarchical omega coefficient (ωH) for the total score was 0.873, whereas the ωHS for the eight subscales ranged from 0.386 to 0.473.

### Subgroup analyses on IIP-32 scores

Subgroup analyses on IIP-32 scores are shown in Table 5. As Table 5 shows, clinical participants' scores were higher than non-clinical participants' scores on all IIP-32 subscales (at least *p* < 0.01). Moreover, clinical participants' scores were higher than non-clinical participants' score on the overall IIP-32 score.

**Table 5:** Subgroup analyses on IIP-32 scores.

	**Clinical sample (*n* = 600)**	**Non-clinical sample (*n* = 623)**	
***IIP-32 Subscales***	**M (*****SD*****)**	**M (*****SD*****)**	***t***
IIP-PA	56.96 (13.19)	54.15 (10.99)	4.03^***^
IIP-BC	50.59 (9.10)	49.17 (6.82)	3.08^**^
IIP-DE	49.92 (8.98)	47.92 (7.03)	4.33^***^
IIP-FG	53.14 (12.40)	49.08 (9.72)	6.36^***^
IIP-HI	55.97 (10.45)	50.96 (8.62)	9.11^***^
IIP-JK	59.95 (10.99)	54.69 (9.43)	8.97^***^
IIP-LM	58.60 (10.88)	54.72 (9.00)	6.78^***^
IIP-NO	60.35 (13.78)	54.89 (11.43)	7.52^***^
IIP-32 total score	57.69 (8.96)	52.45 (7.95)	10.79^***^

IIP-32, Inventory of Interpersonal Problems-32; PA, IIP-32 Domineering/Controlling subscale; BC, IIP-32 Vindictive/ Self-centered subscale; DE, IIP-32 Cold/ Distant subscale; FG, IIP-32 Socially Inhibited/Avoidant subscale; HI, IIP-32 Non-assertive subscale; JK, IIP-32 Overly Accommodating/Exploitable subscale; LM, IIP-32 Self-sacrificing/Overly nurturant subscale; NO, IIP-32 Intrusive/Needy subscale;

** p < 0.01;

*** *p < 0.001*.

### Construct validity of the IIP-32

To provide data on the construct validity of the IIP-32, correlations between the IIP-32 and other questionnaires were examined in a sub-sample of 897 participants (*N* = 601 clinical and *N* = 296 healthy participants) who also completed measures of self-esteem (RSES) and psychological functioning (OQ-45). Table 6 shows the correlations between the scores on IIP-32 (both the total score and eight subscales) and those on the RSES and the OQ-45 for the clinical and non-clinical samples separately. The following theoretically predicted correlations were found for both samples: (a) high interpersonal difficulties (both the overall and the eight-domain scores) were associated with lower self-esteem levels; and (b) high interpersonal difficulties (both the overall and the eight-domain scores) were associated with higher dysfunctional psychological functioning. Only for the non-clinical sample was the Vindicative/Self-Centered subscale not significantly related with self-esteem levels.

**Table 6:** Correlations between IIP-32 subscales and other questionnaires (RSES and OQ-45) for the clinical (*n* = 601) and non-clinical samples (*n* = 296).

	**IIP tot**	**PA**	**BC**	**DE**	**FG**	**HI**	**JK**	**LM**	**NO**
**CLINICAL SAMPLE**									
RSES	−0.471	−0.136	−0.166	−0.333	−0.428	−0.420	−0.293	−0.272	−0.107
OQ-45	0.538	0.239	0.233	0.319	0.425	0.503	0.307	0.263	0.207
**NON-CLINICAL SAMPLE**									
RSES	−0.408	−0.135	−0.096	−0.205	−0.401	−0.370	−0.321	−0.164	−0.300
OQ-45	0.582	0.322	0.284	0.330	0.524	0.435	0.444	0.277	0.373

*IIP tot, Inventory of Interpersonal Problems – total score; PA, IIP Domineering/Controlling Subscale; BC, IIP Vindictive/Self-Centered Subscale; DE, IIP Cold/Distant Subscale; FG, IIP Socially Inhibited/Avoidant Subscale; HI, IIP Non-assertive Subscale; JK, IIP Overly Accommodating/Exploitable Subscale; LM, IIP Self-Sacrificing/Overly Nurturant Subscale; NO, IIP Intrusive/Needy Subscale; RSES, Rosenberg Self-Esteem Scale; OQ-45, Outcome Questionnaire*.

### Associations of the IIP-32 subscales with core symptoms of eating disorders

A series of hierarchical regression analyses were conducted where eating disorder core symptoms, as measured by EDRC and GPMC composite scales of the EDI-3, were regressed on all IIP-32 subscales (see Table 7) and IIP total score (data not shown in the table) for the group of patients with eating disorder (*N* = 182). Control variables (age and gender) were included in the first step, followed by the eight IIP-32 subscales or the total score in the second step. In all models, gender (0 = males, 1 = females) was related to EDRC: women report higher risk for an eating disorder. The Cold/Distant, Socially Inhibited, Nonassertive, and Self-Sacrificing subscales were positively related to GPMC composite. The IIP-32 total score was related to both EDRC and GPMC composites (= 0.245, *t* = 3.394, *p* < 0.01 and = 0.539, *t* = 8.320, *p* < 0.001 for EDRC and GPMC, respectively). Both significant associations were positive for the two EDI-3 composite scales.

**Table 7:** Hierarchical multiple regression analyses with IIP-32 total score predicting eating disorders core symptoms (*n* = 182 clinical subjects with eating disorders).

**Criterion**	**Step**	***Adj R*^2^**	***F*_*change*_**	***p***	**Predictors**	**β**	***t***	***p***
EDRC	1	0.021	2.900	0.058	Gender	0.175	2.369	0.019
					Age	−0.018	−0.247	0.805
	2	0.049	1.672	0.109	Gender	0.173	2.323	0.021
					Age	0.008	0.102	0.919
					IIP-PA	0.061	0.678	0.499
					IIP-BC	0.063	0.650	0.516
					IIP-DE	−0.006	−0.055	0.956
					IIP-FG	0.079	0.810	0.419
					IIP-HI	0.018	0.174	0.862
					IIP-JK	0.034	0.328	0.743
					IIP-LM	0.161	1.644	0.102
					IIP-NO	0.045	0.468	0.640
GPMC	1	0.051	5.300	0.006	Gender	0.140	1.809	0.072
					Age	−0.194	2.504	0.013
	2	0.389	11.905	<0.001	Gender	0.117	1.846	0.067
					Age	−0.105	−1.596	0.113
					IIP-PA	0.003	0.041	0.967
					IIP-BC	0.026	0.311	0.757
					IIP-DE	0.316	3.485	0.001
					IIP-FG	0.171	2.071	0.040
					IIP-HI	0.181	2.031	0.044
					IIP-JK	0.047	0.541	0.589
					IIP-LM	0.192	2.271	0.025
					IIP-NO	−0.017	−0.209	0.835

*EDRC, Eating Disorder Risk Composite; GPMC, General Psychological Maladjustment Composite; IIP, Inventory of Interpersonal Problems; PA, IIP Domineering/Controlling Subscale; BC, IIP Vindictive/Self-Centered Subscale; DE, IIP Cold/Distant Subscale; FG, IIP Socially Inhibited/Avoidant Subscale; HI, IIP Non-assertive Subscale; JK, IIP Overly Accommodating/Exploitable Subscale; LM, IIP Self-Sacrificing/Overly Nurturant Subscale; NO, IIP Intrusive/Needy Subscale*.

## Discussion

The current study was the first to examine the cross-cultural generalizability of the psychometric properties of the Italian version of IIP-32 in both a clinical and non-clinical sample.

Regarding the first aim of this study, we found that the eight-factor CFA solution did not provide an acceptable fit of the data, whereas the eight-factor ESEM solution fits the data much better. Previous factor analyses on the IIP-32 did not report satisfactory findings, suggesting a modest fit with the data (Vanheule et al., 2006; McEvoy et al., 2013). Our results suggest that the ESEM solution is superior to the CFA solution and provides an important test for the adequacy of the ESEM model. When we consider the number of cross-loadings which were statistically different from zero in ESEM solution, it is not surprising that the CFA solution, which constrained these loadings to zero, had a substantially worse fit.

It is also noteworthy that factors were more distinct in the ESEM solution than in the CFA solution: correlations among factors are likely to be higher in CFA solution than ESEM solution whenever there are substantial cross-loadings. However, our results showed that six factor loadings tend to be modest, and 10 items showed cross-loadings higher than 0.30, especially between Overly Accommodating and Non-assertive factors, and between Socially Inhibited and Cold/Distant. This finding is consistent with the Spanish study on the IIP (Salazar et al., 2010), which reported that three factors seem to be defined by a combination of two factors (i.e., Non-assertive and Overly Accommodating, Cold/Distant and Socially Inhibited, Domineering and Intrusive). In sum, our results on the factorial structure of the IIP-32 are not conclusive and it seems that the IIP-32 structure can vary according to cultural and clinical characteristics of the study's participants. Further research with different clinical samples is warranted to support the cross-cultural generalizability of the IIP-32. Moreover, future research should investigate the bifactor structure of the IIP-32, in order to test whether a single, general score, and/or subscale scores should be computed.

The current study was the first to test the measurement invariance of the IIP-32 across clinical and non-clinical groups by the ESEM approach. The eight-factor ESEM model showed a good fit with data both in clinical and non-clinical groups. Strong measurement invariance across groups was supported (with regard to full configural, metric, scalar invariance, and partial strict measurement invariance), highlighting an absence of differential item functioning between the clinical and non-clinical groups. Moreover, this finding supports that the observed differences in means between clinical and non-clinical groups were due to mean differences in factors. Only one previous study (McEvoy et al., 2013) on a different version of the IIP-32 (Barkham et al., 1996) tested a measurement model with two clinical samples, and supported its configural, metric, and scalar invariance. Our findings extend these previous results by supporting the measurement invariance of the IIP-32 by Horowitz et al. (2000) across clinical and non-clinical groups in Italy.

Consistently with the second study's hypothesis, our results showed that the Italian version of the IIP-32 differentiated non-clinical participants and clinical participants on the total score and all subscales, suggesting that people who reported a clinical condition are more likely to have interpersonal problems, accordingly with previous research on the psychological characteristics of patients with eating problems and obesity as well (Hartmann et al., 2010; Lo Coco et al., 2012).

Furthermore, the omega coefficients for the subscales of the IIP-32 were all above 0.77, indicating a moderate to high internal consistency. However, it should be noted that the values of ωHS for the eight subscales were low, suggesting that an extensive proportion of explained total score variance can be attributed to an overall factor after accounting for the eight specific factors.

Previous studies on the internal consistency of the IIP-32 used the Cronbach's alpha coefficients and reported unsatisfactory values for some of the eight factors (Vanheule et al., 2006; Salazar et al., 2010). Our results suggest the importance of the variance partitioning pattern when examining the reliability of the IIP-32 latent factors.

In accordance with the study's hypothesis, the construct validity of the Italian IIP-32 was confirmed through significant correlations between IIP-32 scores and self-reported measures of psychological functioning and self-esteem.

Finally, our results showed that the IIP-32 scores were associated with two EDI-3 composite scores in patients with eating disorders. More specifically, the IIP-32 overall score was associated with both the EDRC and GPMC scores, whereas the Self-sacrificing, Cold/Distant, Non-assertive and Socially inhibited subscales of the IIP-32 were related to core GPMC symptoms. Previous research showed that patients with anorexia and bulimia nervosa reported more severe interpersonal distress in the areas of social inhibition and non-assertiveness (Hartmann et al., 2010), and that patients with BED reported higher submissiveness scores than non-clinical samples (Tasca et al., 2012). Furthermore, our finding is consistent with previous studies (McEvoy et al., 2013) which showed that interpersonal styles reflecting social awkwardness and an excess of friendly submissiveness were consistently associated with a general risk of developing or having an eating disorder and with general psychological maladjustment related to eating disorders (such as perfectionism and low self-esteem) within a clinical sample of patients with anorexia nervosa, bulimia nervosa, and Unspecified Feeding or Eating Disorder. This study extended these findings by demonstrating that the IIP dimensions characterized by low interpersonal dominance also explained a general maladjustment associated with eating symptoms within a more broadly ED clinical sample that also included patients with BED/NED.

Taken together, these findings seem to provide further support to the usefulness of the interpersonal model of eating disorders (Arcelus et al., 2013; Ivanova et al., 2015; Lo Coco et al., 2016) as well as the importance of delivering an interpersonal therapy (i.e., IPT) targeted at these interpersonal styles with these clinical subgroups.

### Strengths and limitations

This study has several limitations. First, an important limitation of this study is the cross-sectional nature of the data and the correlational research design, which limit their utility for causal inferences about relationships among the constructs. Longitudinal studies would further clarify the nature of the association between interpersonal difficulties and core eating disorder symptoms. Secondly, the clinical sample was heterogeneous in terms of eating disorder diagnosis and was comprised almost entirely of women. However, previous studies evidenced that interpersonal profiles of patients at intake did not demonstrate significant differences across different subgroups of eating disorders (Hartmann et al., 2010). Further research on the characteristics of interpersonal profiles with specific subgroups of eating disorders is needed in order to explore the role of interpersonal problems in eating behaviors (Hopwood et al., 2007).

Notwithstanding these limitations, this study has the strength to evaluating, for the first time, the psychometric properties of the Italian version of the IIP-32 on two large clinical and non-clinical samples.

## Author contributions

GL and SG designed and performed the design of the study and conducted the literature searches. LS, VO, CD, and SG provided the acquisition of the data and undertook the statistical analyses. GL, LS, SG, GP, and GM wrote the first draft of the manuscript. All authors significantly participated in interpreting the results, revising the manuscript, and approved its final version.

### Conflict of interest statement

The authors declare that the research was conducted in the absence of any commercial or financial relationships that could be construed as a potential conflict of interest. The reviewer, AS, and handling Editor declared their shared affiliation.
